# Alterations in Mesenteric Lymph Node T Cell Phenotype and Cytokine Secretion are Associated with Changes in Thymocyte Phenotype after LP-BM5 Retrovirus Infection

**DOI:** 10.1080/17402520500303339

**Published:** 2005-12

**Authors:** Maria C. Lopez, Ronald R. Watson

**Affiliations:** Health Promotion Sciences Enid and Mel Zuckerman College of Public Health The University of Arizona Tucson AZ 85724 USA

## Abstract

In this study, mouse MLN cells and thymocytes from advanced stages of LP^-^BM5 retrovirus infection were studied. A decrease in the percentage of IL-7^+^ cells and an increase in the percentage of IL-16^+^ cells in the MLN indicated that secretion of these cytokines was also altered after LP-BM5 infection. The percentage of MLN T cells expressing IL-7 receptors was significantly reduced, while the percentage of MLN T cells expressing TNFR-p75 and of B cells expressing TNFR-p55 increased. Simultaneous analysis of surface markers and cytokine secretion was done in an attempt to understand whether the deregulation of IFN-Υ secretion could be ascribed to a defined cell phenotype, concluding that all T cell subsets studied increased IFN-Υ secretion after retrovirus infection. Finally, thymocyte phenotype was further analyzed trying to correlate changes in thymocyte phenotype with MLN cell phenotype. The results indicated that the increase in single positive either CD4^+^CD8^-^  or CD4^-^ CD8^+^ cells was due to accumulation of both immature (CD3^-^ ) and mature (CD3^+^) single positive thymocytes. Moreover, single positive mature thymocytes presented a phenotype similar to the phenotype previously seen on MLN T cells. In summary, we can conclude that LP-BM5 uses the immune system to reach the thymus where it interferes with the generation of functionally mature T cells, favoring the development of T cells with an abnormal phenotype. These new T cells are activated to secrete several cytokines that in turn will favor retrovirus replication and inhibit any attempt of the immune system to control infection.


*Clinical & Developmental Immunology*, December 2005; 12(4): 249–257

Taylor & Francis

Taylor & Francis Group

### Alterations in mesenteric lymph node T cell phenotype and cytokine secretion are associated with changes in thymocyte phenotype after LP-BM5 retrovirus infection

MARIA C. LOPEZ & RONALD R. WATSON

*Health Promotion Sciences, Enid and Mel Zuckerman College of Public Health, The University of Arizona, Tucson, AZ, 85724, USA*

**Abstract**

In this study, mouse MLN cells and thymocytes from advanced stages of LP-BM5 retrovirus infection were studied. A decrease in the percentage of IL-7$^+$ cells and an increase in the percentage of IL-16$^+$ cells in the MLN indicated that secretion of these cytokines was also altered after LP-BM5 infection. The percentage of MLN T cells expressing IL-7 receptors was significantly reduced, while the percentage of MLN T cells expressing TNFR-p75 and of B cells expressing TNFR-p55 increased. Simultaneous analysis of surface markers and cytokine secretion was done in an attempt to understand whether the deregulation of IFN-γ secretion could be ascribed to a defined cell phenotype, concluding that all T cell subsets studied increased IFN-γ secretion after retrovirus infection. Finally, thymocyte phenotype was further analyzed trying to correlate changes in thymocyte phenotype with MLN cell phenotype. The results indicated that the increase in single positive either CD4$^+$CD8$^-$ or CD4$^-$CD8$^+$ cells was due to accumulation of both immature (CD3$^-$) and mature (CD3$^+$) single positive thymocytes. Moreover, single positive mature thymocytes presented a phenotype similar to the phenotype previously seen on MLN T cells. In summary, we can conclude that LP-BM5 uses the immune system to reach the thymus where it interferes with the generation of functionally mature T cells, favoring the development of T cells with an abnormal phenotype. These new T cells are activated to secrete several cytokines that in turn will favor retrovirus replication and inhibit any attempt of the immune system to control infection.

**Keywords:** *T cell differentiation, IFN-γ, TNF receptors, murine AIDS*

### Introduction

Retrovirus infection, especially HIV infection has proven to have devastating effects on humans' immune system, where practically every immune cell is involved in a hopeless counterattack against the virus (Fauci 1988; Stanley et al. 1993; Su et al. 1995). Similar findings are emerging on the effect of simian immunodeficiency virus on monkeys (Neben et al. 1999; Rosenzweig et al. 2000) and of feline immunodeficiency virus on cats (Woo et al. 1997; Johnson et al. 1998). Although there are species-specific differences between the approaches that different retroviruses use to invade different hosts, it appears from the above observations that all retroviruses finally reach the thymus and interfere with T cell differentiation. The murine leukemia retrovirus LP-BM5 has been used by several groups in an attempt to model human infection (Chattopadhyay et al. 1989). LP-BM5 retrovirus induces generalized immunodeficiency characterized by lymphoadenopathy, splenomegaly, and hypergammaglobulinemia (Chattopadhyay et al. 1989). Even though LP-BM5 favors the proliferation of CD4$^+$ cells, instead of inducing their depletion as HIV does, it renders them anergic and therefore, useless to protect the host (Muralidhar et al. 1992). Furthermore, it has been demonstrated that LP-BM5 also reaches the thymus and depletes it of double positive CD4CD8 cells (Lopez et al. 1992a,b, 1993; de Leval et al. 1995; Colombi et al. 1994) as has been shown for HIV in the elegant experiments using SCID mice reconstituted with neonatal thymus that were

Correspondence: Ronald Ross Watson, Division of Health Promotion Sciences, Mel and Enid Zuckerman Arizona College of Public Health, University of Arizona, 1501 N. Campbell Avenue, P.O. Box 245155, Tucson, AZ 85724-5155, USA. Tel: 520 626 2850. Fax: 520 626 6093. E-mail: rwatson@email.arizona.edu

ISSN 1740-2522 print/ISSN 1740-2530 online © 2005 Taylor & Francis
DOI: 10.1080/17402520500303339

later on infected with the virus (Stanley et al. 1993; Bonyhadi and Kaneshima 1997).

Advanced stages of LP-BM5 infection in mice are characterized as in human retrovirus infection, by a lack of resistance to pathogens and opportunistic pathogens (Chui and Owen 1994). The finding that retrovirus-infected mice could become infected with *Cryptosporidium parvum* a gut parasite, was a starting point for an interest in how LP-BM5 infection was altering gut mucosal immunity (Darban et al. 1991). Further studies clearly indicated that while mesenteric lymph nodes (MLN) increased in size and cell numbers the opposite was the case for Peyer's patches and the gut lamina propria (Lopez et al. 1992c). Moreover, these findings were associated with a paucity of IgA plasma cells in the gut lamina propria (Lopez et al. 1992c). Further studies indicated that neither T nor B cells from the MLN could preferentially migrate to the gut lamina propria after retrovirus infection (Lopez et al. 1995, 2002). Moreover, MLN T cells from LP-BM5 infected mice presented a completely abnormal phenotype (Lopez et al. 2002). Several studies have also shown that retrovirus-infected mice secrete inflammatory cytokines like IFN-γ, TNF-α and IL-4 (Gazzinelli et al. 1992; Huang et al. 1993). It has been considered that the increased level of cytokines is either the result of an immune response against the retrovirus or the consequence of LP-BM5 infection modulating the immune response (Uehara et al. 1994).

In this study, the attention was focused on the effect of LP-BM5 infection on MLN cells and thymocytes. Firstly, the ability of MLN T and B cells to express cytokine receptors for IL-7 and TNF-α was studied since both cytokines are involved in T and B cell proliferation, and TNF-α is also involved in the induction of programmed cell death. Secondly, a simultaneous evaluation of cell phenotype and cytokine secretion was done in an attempt to understand whether the deregulation of cytokine secretion could be ascribed to a defined cell phenotype especially for IFN-γ. Thirdly, alterations in IL-7 and IL-16 secretion after LP-BM5 retrovirus infection were studied for the first time. Lastly, thymocyte phenotype was further analyzed in an attempt to correlate changes in thymocyte phenotype with MLN cell phenotype.

### Materials and methods

### *Mice and retrovirus infection*

Female C57BL/6 mice 4 weeks old were obtained from the National Cancer Institute (Frederick, MD). They were kept for 1 week to allow adjustment to the new environment before they were infected with LP-BM5 murine leukemia virus. The LP-BM5 virus inoculum induced the disease with a time course as previously described (Lopez et al. 2002). Infection was allowed for 16 weeks, only mice showing similar level of infection—generalized lymph node enlargement—were studied.

### *Thymus and mesenteric lymph node cell isolation*

Thymi and MLN were pressed through a cell strainer (Falcon 2340; BD Biosciences, San Jose, CA) to release single cells. Cells were collected on RPMI 1640 containing 10% FCS and antibiotics. Cells were washed in medium and counted. Then, cells were ready either for immediate staining or for culture to measure cytokines.

### *Lymphocyte culture for intracellular cytokine and surface marker simultaneous determination*

Isolated lymphocytes in RPMI 1640/10% FCS, and antibiotics were cultured in 24 well plates in the presence of PMA (50 ng/ml), ionomycin (500 ng/ml) and 10μg/ml Brefeldin A (Epicentre Technology Corp, Madison, WI) for 5 h as described previously (López and Holmes 2005). At the end of the incubation period, cells were resuspended in their wells and transferred to a 96 well V-bottom plate to perform the staining. Plates were spun down, vortexed, and 100 μl of PBS/2%BSA/0.01% Na azide, supplemented with Brefeldin A, were added to each well. Cells were fixed with 100 μl of 4% paraformaldehyde (pH 7.4) per well and the plate was incubated on ice for 20 min. Plates were spun down and cells were resuspended in PBS/2%BSA/0.5% saponin/0.01% Na azide, and incubated in the cold for another 20 min, to facilitate pore formation. Anti-CD16/32 (2.4G2) was added as blocking reagent to the permeabilization reagent. At the end of the incubation period, fluorochrome-conjugated antibodies that recognize mouse cytokines were added. Plates were incubated in the cold and darkness for 30 min. Then, they were spun down, washed twice with PBS/2%BSA/0.5% saponin/0.01% Na azide, and once with PBS/2%BSA/0.01% Na azide. Cells were resuspended in PBS/2%BSA/0.01% Na azide, and antibodies recognizing specific surface markers were added, and plates were incubated in the cold and darkness for another 30 min. Afterwards, plates were spun down and the cells were washed with PBS/2%BSA/0.01% Na azide. Finally, cells were resuspended in 2% paraformaldehyde (pH 7.4), stored in darkness at 4°C, until analyzed in a FACStar Plus (BD Biosciences) that included Cellquest software. Samples were always run within 24 h after the staining was finished.

### *Antibodies used in flow cytometric staining*

The following directly conjugated anti-mouse antibodies were used: FITC- and Cychrome-anti-CD3, FITC-anti-CD44, FITC- and PE-anti-CD90.2, FITC-DX5, PE-anti CD120a (TNFR-p55), PE-antiCD120b

(TNFR-p75), PE-anti-IFN-γ, and purified anti-CD16/32 (2.4G2) from BD Pharmingen (San Diego, CA); FITC-, PE- and TC-anti-CD4, TC-anti-γδ-TCR, FITC- PE- and TC- anti-CD8α, FITC-anti-CD19, PE-anti CD62L, PE-anti-IL-2, PE-anti-IL-4, PE-anti-TNF-α, and PE-anti IL-16 from Caltag (Burlingame, CA); and PE-anti-CD127 (IL-7R) from eBioscience (San Diego, CA). Goat anti-mouse IL-7 from R&D systems (Minneapolis, MN) monoclonal antibody was conjugated with FITC in our lab as described previously (López and Holmes 2005).

### *Statistical analysis*

Data from retrovirus infected and uninfected mice were compared using the two-tailed Student's *t* test included in Excel software.

### Results

### *Expression of IL-7R and of TNF-R p55 and TNF-R p75 on MLN B and T cells of retrovirus infected mice*

As in previous reports, data presented in this study analyzed mice at a very advanced stage of LP-BM5 retrovirus infection (Lopez et al. 1992c, 2002). These mice as those studied before presented an increased proportion of $CD3^+$ cells, mainly $CD4^+$ (Figure 1A). Accordingly, there was decreased proportion of B cells ($CD19^+$ cells), as described earlier (Lopez et al. 1992c). IL-7 is involved in proliferation and differentiation of immature T and B cells (Hofmeister et al. 1999). Cells responding to IL-7 express the IL-7 receptor, CD127 (Hofmeister et al. 1999). The percentage of MLN cells expressing CD127 (IL-7 receptor) was significantly reduced in retrovirus-infected mice due to the dramatic decrease in the percentage of $CD3^+$ cells expressing the receptor (Figure 1B). TNF-α is known as an inflammatory cytokine secreted in response to infection. TNF-α binds to its receptors TNFR-p55 and TNFR-p75 that deliver signals triggering either cell proliferation or programmed cell death (Chan et al. 2000). The percentage of B cells expressing CD120a (TNFR-p55) increased in retrovirus-infected animals, as did the percentage of T cells expressing CD120b (TNFR-p75) (Figure 1B).

### *Cytokine secretion profile of MLN cells from retrovirus infected mice*

As shown before using different methods, retrovirus infection is associated with increased secretion of several cytokines (Gazzinelli et al. 1992; Huang et al. 1993). Increased percentages of MLN cells secreting IL-2, IL-4, TNF-α and IFN-γ were observed in agreement with previous data obtained with different techniques (Figure 2A) (Huang et al. 1993). Interestingly, as previously observed in retrovirus-infected humans, the

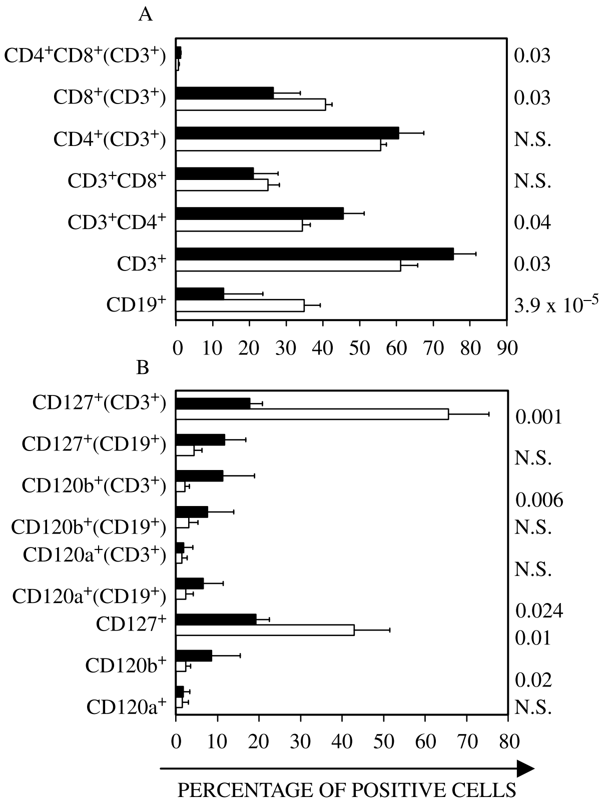

Figure 1. MLN cells phenotype. MLN cells were stained and their phenotype was analyzed using flow cytometry. The percentage of B ($CD19^+$), T ($CD3^+$) and T cell subsets were studied in MLN cells from normal and retrovirus infected mice (A). The expression of IL-7R (CD127) and TNF-R p55 (CD120a) and TNF-R p75 (CD120b) on T and B cells was also analyzed (B). The exact level of signification using the two-tailed Student's *t* test is shown for every marker. Open bars: control mice; filled bars murine AIDS infected mice. $CD3^+CD4^+$ stands for double positive cells, while $CD4^+(CD3^+)$ means the percentage of $CD4^+$ cells within the CD3 population.

percentage of $IL\text{-}16^+$ cells was increased (Center et al. 2000; Cruikshank et al. 2000) and it was found that $CD19^+$ cells were responsible for IL-16 secretion (Figure 2B). On the contrary, the proportion of IL-7 secreting cells decreased mainly due to a change in $CD90^-$ $CD4^+$ cells ability to secrete IL-7. IL-2 and TNF-α secretion increased after retrovirus infection due to the increased ability of $CD3^+$ $CD4^-$ cells from infected mice to secrete both cytokines (Figure 2B). In addition, fewer DX5 cells from infected mice secreted IL-4.

As shown in Figure 3, after gating on several T cell subsets, including $γδ\text{-}TCR^+$ and $CD90^-CD4^+$, an increase in the percentage of cells secreting IFN-γ was observed. Flow cytometry plots indicated that while in normal mice $CD90^{high+}$ cells are mainly responsible for IFN-γ secretion, in retrovirus infected mice $CD90^{dim+}$ and $CD90^-$ cells also contribute to IFN-γ secretion (Figure 4B). In addition, $CD4^+$ cells that in normal mice showed a negligible contribution to the number of cells secreting IFN-γ sharply increase after

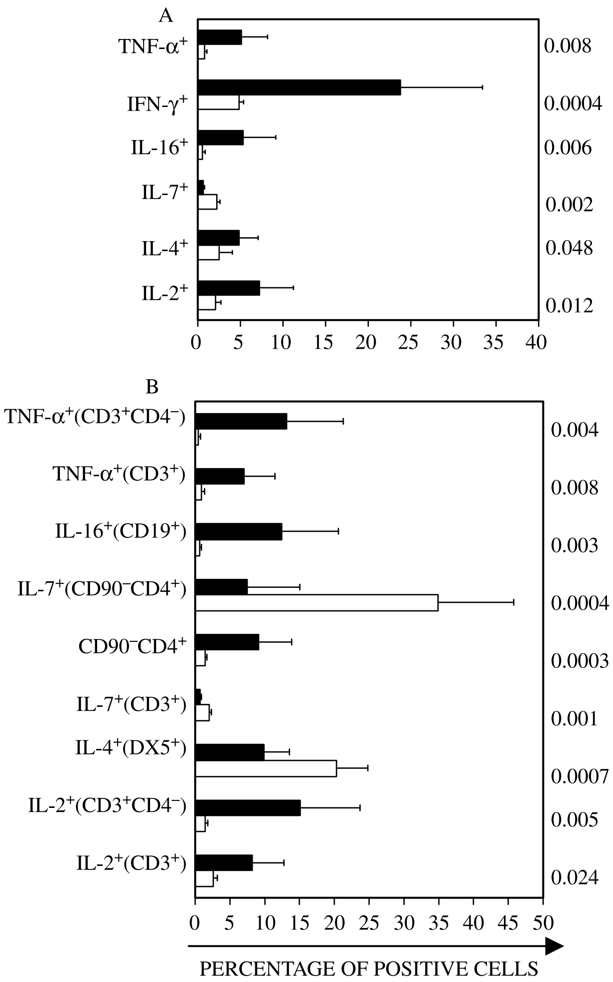

Figure 2. MLN cells cytokine secretion profile. MLN cells were cultured, fixed, permeabilized, stained and analyzed as described in "Materials and methods" and the percentage of cells secreting cytokines was studied (A). After gating on T, B or NK cell markers the percentage of cells secreting cytokines was analyzed (B). The exact level of signification using the two-tailed Student's *t* test is shown for every marker. Open bars: control mice; filled bars murine AIDS infected mice.

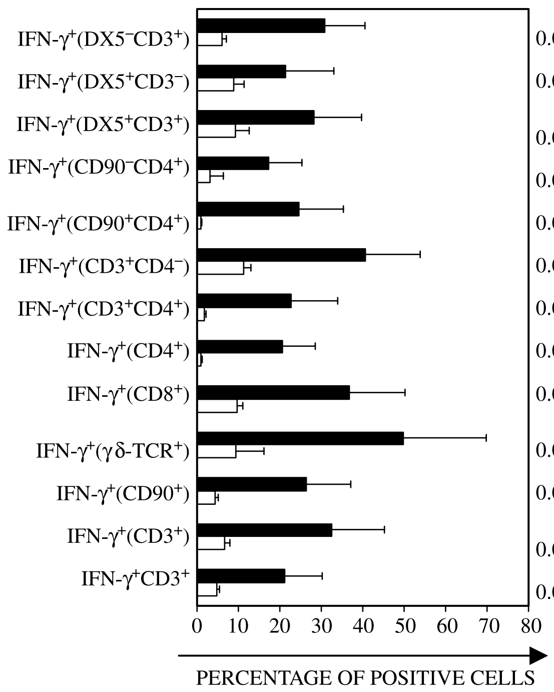

Figure 3. MLN cells secreting IFN-γ. MLN cells were cultured as mentioned in Figure 2 and after gating on different T cell and NK cell markers the percentage of cells secreting IFN-γ was calculated. The exact level of signification using the two-tailed Student's *t* test is shown for every marker. Open bars: control mice; filled bars murine AIDS infected mice.

retrovirus infection (Figure 4C). Furthermore, while in normal animals $CD8^+$ cells seemed to be the main source for IFN-γ secretion, their contribution becomes less significant after retrovirus infection (Figure 4D).

*Changes in surface markers expression on thymocytes of retrovirus infected mice*

It has been shown that retrovirus infection alters T cell differentiation in humans, monkeys and cats (Stanley et al. 1993; Su et al. 1995; Woo et al. 1997; Johnson et al. 1998; Neben et al. 1999; Rosenzweig et al. 2000). In addition, studies in mice infected with LP-BM5 retrovirus have shown similar results characterized by a marked reduction in the percentage of $CD4^+CD8^+$ thymocytes (Lopez et al. 1992a,b, 1993; Colombi et al. 1994; de Leval et al. 1995). The present study has confirmed preliminary data indicating the increase in the percentage of single positive either $CD4^+CD8^-$ or $CD4^-CD8^+$ cells, and has also shown increase in the percentage of double negative cells (Figures 5A, 6A and D). Moreover, a decrease in the total number of thymocytes in LP-BM5 infected mice ($0.24 \times 10^8 \pm 0.07 \times 10^8$ vs. $1.3 \times 10^8 \pm 0.17 \times 10^8$, $p = 4.88 \times 10^{-5}$) was also observed. The percentage of $CD4^+CD8^-$ cells represented a 10 times increase over the normal values and the percentage of $CD4^-CD8^+$ cells represented a 15 times increase. By using three colors staining and gating on single positive populations it was shown that the percentage of cells expressing CD3 had significantly diminished, suggesting the simultaneous accumulation of immature and mature single positive cells in the thymus (Figure 5A, 6B, C, E and F).

Taking into account the expansion of $CD4^+CD90^{--}CD3^+$ cells observed in the periphery (Holmes et al. 1990; Lopez et al. 2002), it became important to analyze whether the distribution of CD90 was also altered on thymocytes. Gating on CD4 or CD8 single positive cells, and studying the expression of CD90 showed no changes for $CD4^-CD8^+$ cells. On the contrary, the percentage of $CD4^+CD8^-$ cells expressing CD90 decreased during retrovirus infection, as did the percentage of $CD4^+CD3^+$ cells expressing CD90 (Figure 5B). Further analysis on the expression of CD3 and CD90 indicated several

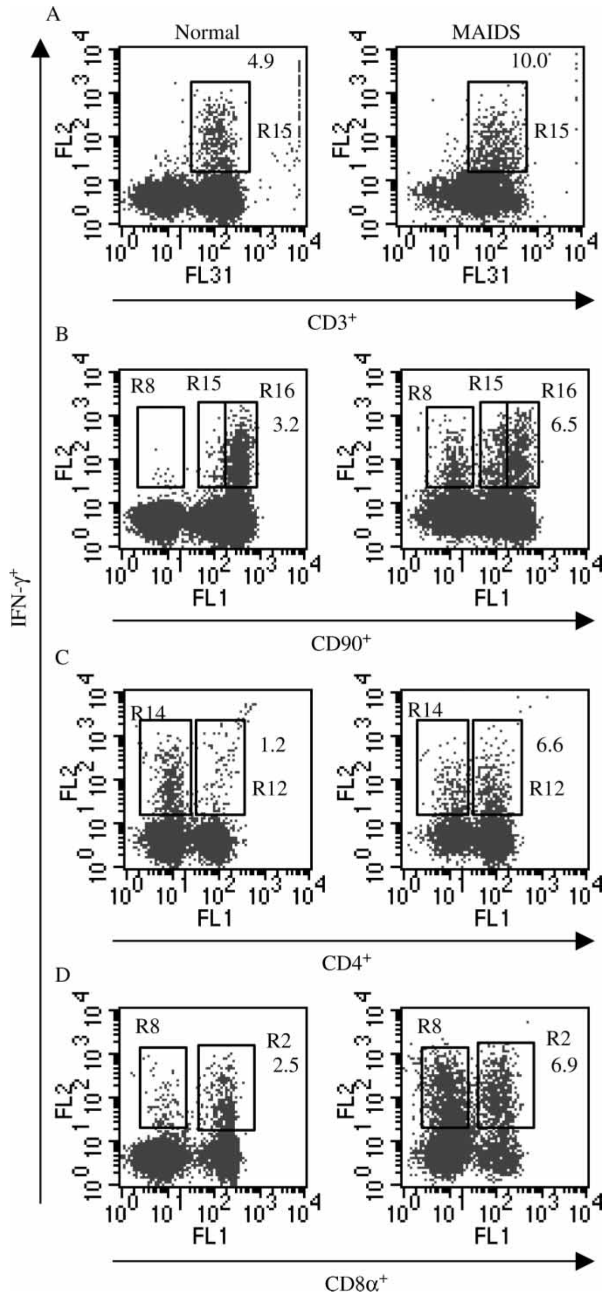

Figure 4. Flow cytometry plots showing MLN cells secreting IFN-γ. Representative plots show different patterns of IFN-γ secreting cells after retrovirus infection. More $CD3^+$ cells secrete IFN-γ after retrovirus infection (A). Not only $CD90^{high+}$ cells secrete IFN-γ in retrovirus infected mice but also $CD90^{low+}$ (R15: 3.73 vs. 0.36%), and $CD90^-$ cells (R8: 4.36 vs. 0.07%) (B). Fewer $CD4^+$ cells secrete IFN-γ in normal mice than in retrovirus infected animals, while the percentage of $CD4^-$ cells secreting IFN-γ may not change in some mice (R14: 4.83 vs. 4.7%) (C). The proportion of $CD8^+$ cells secreting IFN-γ nearly triples, while the proportion of $CD8^-$ cells secreting IFN-γ increases nearly 25 times (R8: 0.70 vs. 17.50) (D).

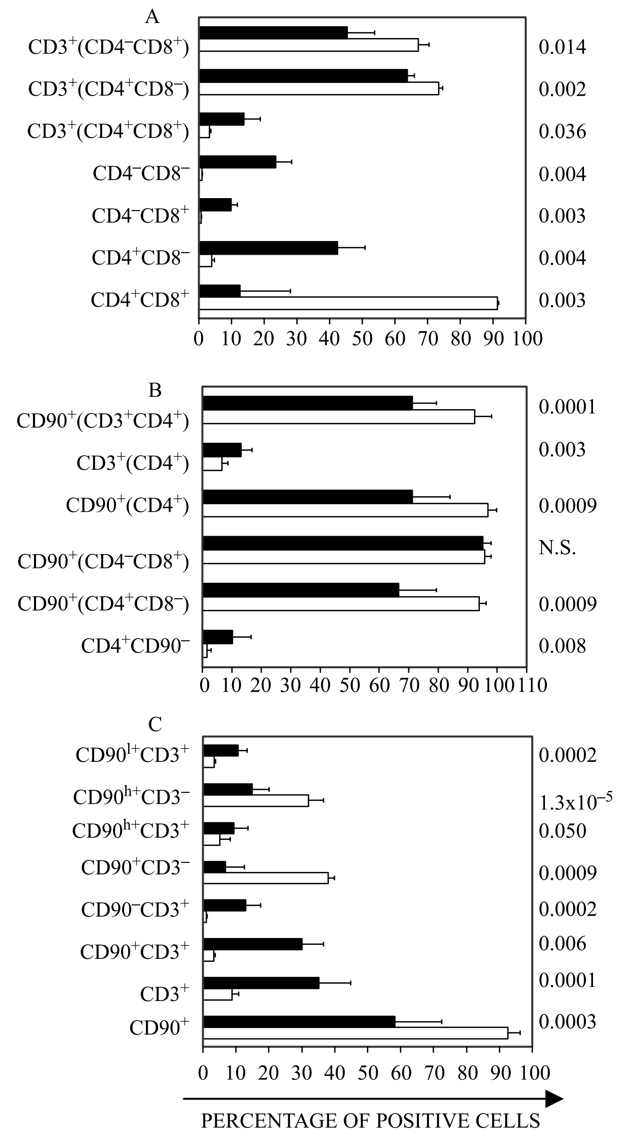

Figure 5. Thymus cells phenotype. Thymocytes were stained and their phenotype was analyzed by flow cytometry. The expression of CD3 on CD4 and CD8 cells is shown in (A). The expression of CD90 was analyzed on CD4 and CD8 cells (B). The double expression of CD3 and CD90 was studied on thymocytes (C). The exact level of signification using the two-tailed Student's *t* test is shown for every marker. Open bars: control mice; filled bars murine AIDS infected mice.

changes. Retrovirus infected mice had less thymocytes expressing CD90 and more thymocytes expressing CD3 (Figure 5C). Concomitant with the increase in the percentage of thymocytes expressing CD3, there was an increase in the percentage of $CD3^+CD90^-$ and $CD3^+CD90^+$ cells. Interestingly, both the percentage of $CD90^{low+}$ $CD3^+$ and $CD90^{high+}CD3^+$ increased, while the percentage of $CD90^{high+}CD3^-$, representing more immature thymocytes decreased (Figure 5C, 6G and H).

It has also been shown that MLN T cells from retrovirus infected mice presented an abnormal expression of CD62L and CD44 (Lopez et al. 2002), raising the question whether or not T cells in the thymus already displayed these markers in a different fashion before migrating to the periphery. Accordingly, after gating on either double positive, or double negative or single positive thymocytes, there was a decrease in the percentage of cells expressing CD62L (Figure 7A). Furthermore, when the same

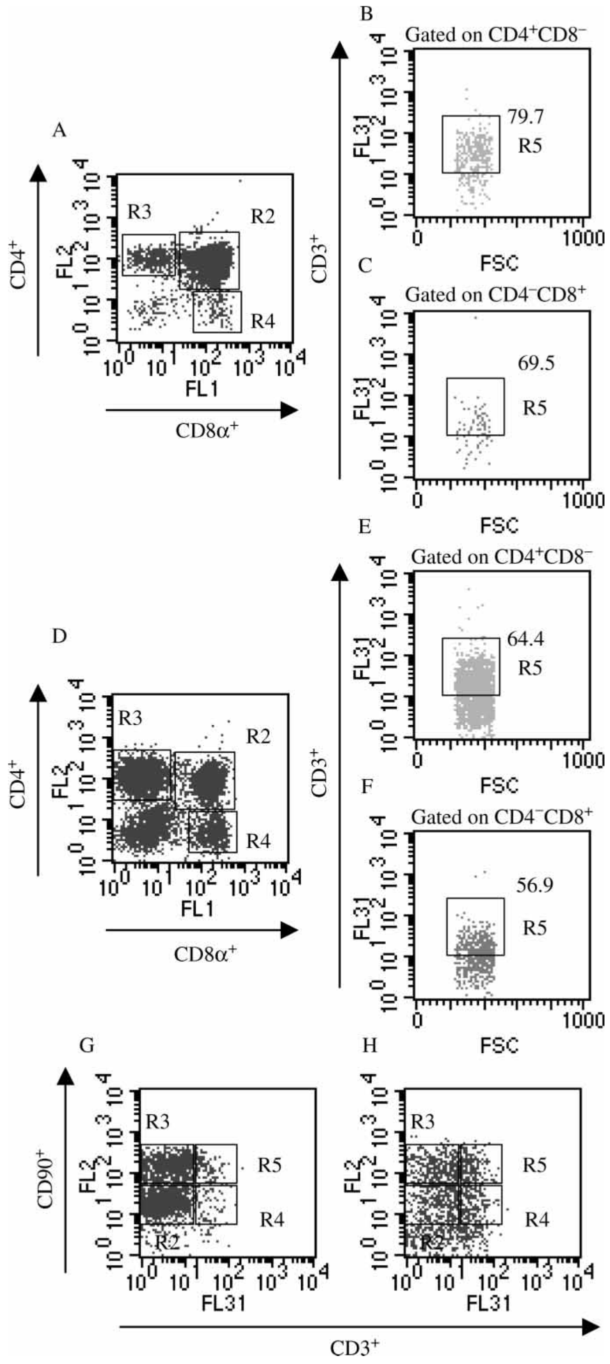

Figure 6. Flow cytometry plots showing thymocytes surface marker expression. The expression of CD4 and CD8 was studied on normal (A) and retrovirus infected (D) mouse thymocytes. R2 represents CD4CD8 double positive cells (normal vs. MAIDS: 91.6 vs. 39.3%), R3 represents $CD4^+$ $CD8^-$ cells (4.9 vs. 30.5%) and R4 represents $CD4^-CD8^+$ cells (1.2 vs. 9.0%). The percentage of $CD3^+$ cells was calculated after gating on $CD4^+CD8^-$ cells (B and E) and after gating on $CD4^-CD8^+$ cells (C and F). Changes in the expression of CD90 and CD3 on thymocytes are shown for representative mice (G normal and H retrovirus infected). R2 represents $CD90^{low+}CD3^-$ (48.7 vs. 22.6%), and R3 represents $CD90^{high+}CD3^-$ (38.2 vs. 24.4%). R4 represents $CD90^{low+}CD3^+$(2.6 vs. 12.3%), and R5 represents $CD90^{high+}CD3^+$(4.4 vs. 12.5%).

analysis was done for the expression of CD44, there was an increase in the percentage of cells expressing CD44 on the gated populations (Figure 7B).

### Discussion

The present study continues previous work aimed to unravel how retrovirus infection in mice disturbs the immune system by deregulating cell proliferation, differentiation and cytokine secretion. In this study the observations on the changes induced by LP-BM5 infection on cytokine secretion were extended to two cytokines that were not studied before in this model: IL-7 and IL-16. IL-7 is known to be a crucial cytokine for early T and B cell development (Hofmeister et al. 1999). Mice lacking either IL-7 or IL-7R α-chain present a severe reduction in the number of cells in the thymus and in peripheral lymphoid organs (Hofmeister et al. 1999; Carvalho et al. 2001). Data obtained in mice at an advanced stage of retrovirus infection indicated that the proportion of $IL\text{-}7^+$ cells diminished in the MLNs of infected mice, as did the percentage of CD3 cells expressing the IL-7 receptor CD127. Studies in HIV infected individuals presented a similar decrease in the expression of IL-7 receptor, especially on CD8 cells, that was associated with lack of cytotoxic function against the retrovirus (Carini et al. 1994; MacPherson et al. 2001). Interestingly, antiretroviral therapy increased IL-7 R expression in patients (Ferrari et al. 1995). Taken together, these observations suggest that retrovirus infection down regulates the host's IL-7 secretion and IL-7 receptor expression to facilitate its own replication. Notwithstanding, human studies have also shown that IL-7 can facilitate HIV replication suggesting that the

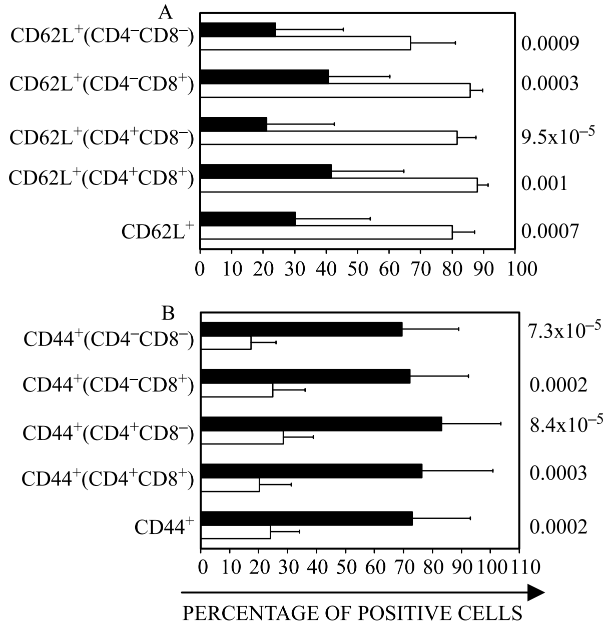

Figure 7. Surface markers expressed on CD4CD8 thymocytes. The expression of CD62L (A) and CD44 (B) was studied on CD4CD8 double positive, single positive and double negatives thymocytes. The exact level of signification using the two-tailed Student's *t* test is shown for every marker. Open bars: control mice; filled bars murine AIDS infected mice.

cytokine can play a protective role for the host and for the virus (Wang et al. 2005).

IL-16 is a cytokine secreted by a wide variety of lymphoid and non-lymphoid cells that was originally known as a T cell-specific chemoattractant factor (Cruikshank et al. 2000). The results presented in this study indicate that B cells are responsible for the increased percentage of IL-16$^+$ cells. Studies in humans infected with HIV indicated that IL-16 can inhibit retrovirus replication and that any drop in IL-16 serum levels can be associated with disease progression (Center et al. 2000; Devadas et al. 2003). Moreover, the possibility of using IL-16 as part of the anti-retroviral treatment in humans has been widely discussed (Devadas et al. 2003). No conclusion can be made up to the ability of mouse IL-16 to inhibit viral replication from the data presented in this report. Besides, it is known that IL-16 binds to its receptor CD4 and preferentially induces the migration of Th1 cells (Lynch et al. 2003). Therefore, it could be hypothesized that an increase in IL-16 levels in the MLN will be associated with an increased migration of CD4 Th1 type cells. It is not clear yet whether IL-16 can have any inhibitory effect on the migration of CD4 Th2 type cells to effector sites. If that were the case, it could be speculated that the retention of CD4 Th2 type cells involved in IgA B cell help in the MLN, and their inability to home to the intestinal lamina propria observed in previous studies; was in part caused by the increased levels of IL-16 (Lopez et al. 2002). Thus, IL-16 increase in retrovirus infected mice would be deleterious for the host since it could interfere with maintenance of protective mechanisms at mucosal sites.

The increase in IFN-γ secretion by lymphoid cells of LP-BM5 infected mice has been extensively investigated (Gazzinelli et al. 1992; Huang et al. 1993; Uehara et al. 1994). In this study, the aim was to analyze whether or not one specific T cell subset was the main responsible for the higher levels of cytokine detected. Simultaneous analysis of surface markers and cytoplasmic IFN-γ indicated that all T cell subsets studied were activated to secrete the cytokine, including CD90$^-$CD4$^+$, CD90$^+$CD4$^+$ and CD8$^+$ MLN cells. These observations in retrovirus infected mice contrast with data from non-infected animals where only a few CD8$^+$ cells and very few CD4$^+$ cells secrete IFN-γ. It has been reported that CD90$^-$CD4$^+$ cells are not active at secreting certain cytokines like IFN-γ in healthy hosts (Cerasoli et al. 2001) nonetheless in retrovirus-infected mice they were actively secreting IFN-γ. Some studies have suggested that CD90$^-$CD4$^+$ cells that proliferate in retrovirus-infected mice are responsible for transmitting the retrovirus after transfer into naïve hosts (Kubo et al. 1992; Masuda et al. 1996). Therefore, retrovirus infection could be responsible for CD90$^-$CD4$^+$ cells activation to secrete cytokines. This unique subset of T cells has been first identified in Peyer's patches of healthy mice (Harriman et al. 1990), in the spleens of LP-BM5 infected mice (Holmes et al. 1990) and in the thymus of Cas-Br-E MuLV infected mice (Cerasoli et al. 2001). It has been suggested that IFN-γ increase in LP-BM5 infected mice may not be the result of a protective immune response mounted by the host; but of a retrovirus induced immune deregulation (Uehara et al. 1994). In fact when mice were infected with the retrovirus and treated with anti-IFN-γ antibody the development of lymphadenopathy, immunodeficiency and increased levels of IgG2a were delayed (Uehara et al. 1994). These findings could explain why CD90$^-$ CD4$^+$ cells only secrete IFN-γ after LP-BM5 infection.

Results obtained in this study showed an increase in the percentage of CD3$^+$ cells expressing TNFR-p75 (CD120b) in the MLN of retrovirus infected mice, that was associated with an increase in the percentage of cells secreting TNF-α, while CD19$^+$ cells expressed higher levels of TNFR-p55 (CD120a). Current knowledge suggests that signaling through TNFR-p55 generally leads to cell death while signaling through TNFR-p75 leads to cell survival although the opposite is also true (Chan et al. 2000; Gonzalez Baseta and Stutman 2000). Based on that information, it could be concluded that higher levels of TNF-α associated with increased expression of CD120b favored the survival of infected T cells in this model of retrovirus infection.

In previous studies, we have shown a decrease in the number of thymocytes after LP-BM5 infection (Lopez et al. 1992a,b) as well as a decrease in the percentage of double positive CD4CD8 thymocytes (Lopez et al. 1992a) and the disappearance of the cortical areas of the thymus 11 weeks after infection (Lopez et al. 1993). As shown by us and by Moutschen's group the diminished percentage of double positive CD4CD8 cells is accompanied by an increase in the percentage of single positive CD4$^+$CD8$^-$ and CD4$^-$CD8$^+$ thymocytes (Lopez et al. 1992a; Colombi et al. 1994; de Leval et al. 1995). The triple staining analysis done in this study allowed us to show that not all single positive cells found in the thymus are mature and therefore express CD3, on the contrary, immature precursors also accumulated. These data suggest that murine retrovirus infection interfered with intrathymic T cell differentiation. Elegant studies on T cell differentiation have suggested two alternative pathways leading to CD4CD8 double positive cells. On one hand, it has been shown that CD4$^+$ thymocytes developed from CD4$^-$CD8$^+$CD3$^-$ thymocytes that represent a transitional intermediate from CD3$^-$CD4$^-$CD8$^-$ thymocytes to CD4$^+$CD8$^+$CD3$^-$ thymocytes (Smith 1987; MacDonald et al. 1988). On the other hand, it has also shown the existence of CD4$^+$CD8$^-$CD3$^-$ thymocytes that can differentiate into CD4$^+$CD8$^+$CD3$^-$ thymocytes (Hugo et al. 1990, 1991; Wu et al. 1991). It is considered that both intermediate stages require different post-selection processing for final development (Petrie et al. 1993). Our data clearly indicated that both intermediates accumulated within the thymus suggesting that the decrease in the percentage of $CD4^+CD8^+$ cells could be the result of the inability of transitional single positive intermediates to differentiate into double positive thymocytes due to retrovirus infection. Other retroviruses have shown similar patterns of alterations in T cell differentiation. Feline immunodeficiency virus induces cortical involution and decrease in the percentage of $CD4^+CD8^+$ thymocytes (Woo et al. 1997). Similar results were obtained after simian immunodeficiency virus infection (Rosenzweig et al. 2000), and when immature $CD3^{--}$ $CD4^+CD8^-$ thymocytes isolated from infected monkeys were examined in the fetal thymus organ culture system, they presented impaired thymopoietic potential (Neben et al. 1999). Furthermore, the HIV-infected SCID-hu mouse system has shown destruction of the cortical area of the thymus, disappearance of $CD4^+CD8^+$ cells and indication that $CD3^-CD4^+CD8^-$ progenitor were destroyed intrathymically after HIV infection (Stanley et al. 1993). Taken together, these data suggest that retroviruses migrate to the host's thymus to interfere with early stages of T cell differentiation. Furthermore, thymocytes that did acquire a mature phenotype either $CD4^+CD8^-CD3^+$ or $CD4^-CD8^+CD3^+$ after LP-BM5 infection also accumulated in the thymus and presented an altered expression of CD44 and CD62L that resembled the expression previously observed on spleen and MLN lymphocytes (Muralidhar et al. 1992; Lopez et al. 2002).

In summary, we can conclude that LP-BM5 uses the immune system to reach the thymus where it interferes with the generation of functionally mature T cells, favoring the development of T cells with an abnormal phenotype. These new T cells are activated to secrete several cytokines that in turn will favor retrovirus replication and inhibit any attempt of the immune system to control infection.

### Acknowledgements

The authors greatly appreciate the skillful assistance of Barb Carolus in the Biotechnology Imaging Facilities, ARL-Biotechnology. This work was supported by NIH grant HL 63667.

### References

Bonyhadi ML, Kaneshima H. 1997. The SCID-hu mouse: An *in vivo* model for HIV-1 infection in humans. Mol Med Today 3:246–253.

Carini C, McLane MF, Mayer KH, Essex M. 1994. Dysregulation of interleukin-7 receptor may generate loss of cytotoxic T cell response in human immunodeficiency virus type 1 infection. Eur J Immunol 24:2927–2934.

Carvalho TL, Mota-Santos T, Cumano A, Demengeot J, Vieira P. 2001. Arrested B lymphopoiesis and persistence of activated B cells in adult interleukin $7^{-/-}$ mice. J Exp Med 194:1141–1150.

Center DM, Kornfeld H, Ryan TC, Cruikshank WW. 2000. Interleukin 16: Implications for CD4 functions and HIV-1 progression. Immunol Today 21:273–280.

Cerasoli DM, Kelsoe G, Sarzotti M. 2001. $CD4^+Thy1^-$ thymocytes with a Th-type 2 cytokine response. Int Immunol 13:75–83.

Chan FK-M, Siegel RM, Lenardo MJ. 2000. Signaling by the TNF receptor superfamily and T cell homeostasis. Immunity 13:419–422.

Chattopadhyay SK, Makino M, Hartley JW, Morse III, HC. 1989. Pathogenesis of MAIDS, a retrovirus induced immunodeficiency disease in mice. In: Wu B-q, Zheng J, editors. Immunodeficient animals in experimental medicine. Basel: Karger. p 12–18.

Chui DW, Owen RL. 1994. AIDS and the gut. J Gastroenterol Hepatol 9:291–303.

Colombi S, Deprez M, de Leval L, Humblet C, Greimers R, Defresne M-P, Boniver J, Moutschen M. 1994. Thymus involvement in murine acquired immunodeficiency (MAIDS). Thymus 23:27–37.

Cruikshank WW, Kornfeld H, Center DM. 2000. Interleukin-16. J Leuk Biol 67:757–766.

Darban H, Enriquez J, Sterling CR, Lopez MC, Chen G, Abbaszadegan M, Watson RR. 1991. Cryptosporidiosis facilitated by murine retroviral infection with LP-BM5. J Infect Dis 164:741–745.

de Leval L, Deprez M, Colombi S, Humblet C, Defresne M-P, Boniver J, Moutschen M. 1995. Morphological changes of thymus in retrovirus-induced murine acquired immunodeficiency syndrome (MAIDS). Path Res Pract 191:506–512.

Devadas K, Zhou P, Tweari D, Notkins AL. 2003. Inhibition of HIV-1 replication by the combined action of anti-gp41 single chain antibody and IL-16. Antiviral Res 59:67–70.

Fauci AS. 1988. The human immunodeficiency virus: Infectivity and mechanisms of pathogenesis. Science 239:617–622.

Ferrari G, King K, Rathbun K, Place CA, Packard MV, Bartlett JA, Bolognesi DP, Weinhold KJ. 1995. IL-7 enhancement of antigen-driven activation/expansion of HIV-1-specific cytotoxic T lymphocyte precursors (CTLp). Clin Exp Immunol 101:239–248.

Gazzinelli RT, Makino M, Chattopadhyay SK, Snapper CM, Sher A, Hugin AW, Morse III, HC. 1992. $CD4^+$ subset regulation in viral infection. Preferential activation of Th2 cells during progression of retrovirus-induced immunodeficiency in mice. J Immunol 148:182–188.

Gonzalez BJ, Stutman O. 2000. TNF regulates thymocyte production by apoptosis and proliferation of the triple negative ($CD3^-CD4^-CD8^-$) subset. J Immunol 165:5621–5630.

Harriman GR, Lycke NY, Elwood LJ, Strober W. 1990. T lymphocytes that express CD4 and the αβ-T cell receptor but lack Thy-1. J Immunol 145:2406–2414.

Hofmeister RA, Khaled R, Benbernou N, Rajnavolgyi E, Muegge K, Durum SK. 1999. Interleukin-7: Physiological roles and mechanisms of action. Cytokine Growth Factor Rev 10:41–60.

Holmes KL, Morse III, HC, Makino M, Hardy RR, Hayakawa H. 1990. A unique subset of normal murine $CD4^+$T cells lacking Thy-1 is expanded in a murine retrovirus-induced immunodeficiency syndrome, MAIDS. Eur J Immunol 20:2783–2787.

Huang DS, Wang Y, Marchalonis JJ, Watson RR. 1993. The kinetics of cytokine secretion and proliferation by mesenteric lymph node cells during the progression to murine AIDS, caused by LP-BM5 murine leukemia virus infection. Reg Immunol 5:325–331.

Hugo P, Waanders GA, Scollay R, Shortman K, Boyd RL. 1990. Ontogeny of a novel $CD4^+CD8^-CD3^-$ thymocyte subpopulation: A comparison with $CD4^-$ $CD8^+$ $CD3^-$ thymocytes. Int Immunol 2:209–218.

Hugo P, Waanders GA, Scollay R, Petrie HT, Boyd RL. 1991. Characterization of immature $CD4^+CD8^-CD3^-$ thymocytes. Eur J Immunol 21:835–838.

Johnson CM, Papadi P, Tompkins WA, Sellon RK, Orandle MS, Bellah JR, Bubenik LJ. 1998. Biphasic thymus response by kittens inoculated with feline immunodeficiency virus during fetal development. Vet Pathol 35:191–201.

Kubo Y, Nakagawa Y, Kakimi K, Matsui H, Iwashiro M, Kuribayashi K, Masuda T, Hiai H, Hirama T, Yanagawa S-I, Ishimoto A. 1992. Presence of transplantable T-lymphoid cells in C57BL/6 mice infected with murine AIDS virus. J Virol 66:5691–5695.

Lopez MC, Colombo LL, Huang DS, Wang Y, Watson RR. 1992a. Modification of thymic cell subsets induced by long-term cocaine administration during a murine retroviral infection producing AIDS. Clin Immunol Immunopathol 65:45–52.

Lopez MC, Colombo LL, Huang DS, Watson RR. 1992b. Alteration of thymic cell subsets by cocaine administration and murine retrovirus infection in protein undernourished mice. Thymus 20:171–181.

Lopez MC, Colombo LL, Huang DS, Watson RR. 1992c. Suppressed mucosal lymphocyte populations by LP-BM5 murine leukemia virus infection producing murine AIDS. Reg Immunol 4:162–167.

Lopez MC, Chen G-J, Colombo LL, Huang DS, Darban HR, Watzl B, Watson RR. 1993. Spleen and thymus cell subsets modified by long-term morphine administration and murine AIDS-II. Int J Immunopharmacol 15:909–918.

Lopez MC, Huang DS, Way DL, Watson RR. 1995. Functional and phenotypic lymphocyte population changes in the mesenteric lymph nodes of murine-AIDS infected mice. Adv Exp Med Biol 371B:1039–1042.

Lopez MC, Huang DS, Watson RR. 2002. Changes in mesenteric lymph node T cell phenotype and B and T cell homing properties after murine AIDS infection. Lymphology 35:76–86.

López MC, Holmes N. 2005. Phenotypical and functional alterations in the mucosal immune system of CD45 exon 9 KO mice. Int Immunol 17:15–25.

Lynch EA, Heijens CAW, Horst NF, Center DM, Cruikshank WW. 2003. Cutting edge: IL-16/CD4 preferentially induces Th1 cell migration: Requirement of CCR5. J Immunol 171:4965–4968.

MacDonald HR, Budd RC, Howe RC. 1988. A $CD3^-$ subset of $CD4^-8^+$ thymocytes: A rapidly cycling intermediate in the generation of $CD4^+8^+$ cells. Eur J Immunol 18:519–523.

MacPherson PA, Fex C, Sanchez-Dardon J, Hawley-Foss N, Angel JB. 2001. Interleukin-7 receptor expression on CD8(+) T cells is reduced in HIV infection and partially restored with effective antiretroviral therapy. J Acq Immun Def Synd 28:454–457.

Masuda A, Makino M, Kasajima T. 1996. Contribution of $Thy\text{-}1^-CD4^+$T cells to the disorganization of lymphoid follicles in retrovirus-induced immunodeficiency syndrome, MAIDS. Clin Immunol Immunopathol 81:253–260.

Muralidhar G, Koch S, Haas M, Swain S. 1992. CD4 T cells in murine acquired immunodeficiency syndrome: Polyclonal progression to anergy. J Exp Med 175:1589–1599.

Neben K, Heidbreder M, Müller J, Marxer A, Petry H, Didier A, Schimp A, Hünig T, Kerkau T. 1999. Impaired thymopoietic potential of immature $CD3^-CD4^+CD8^-$ T cell precursors from SIV-infected rhesus monkeys. Int Immunol 11:1509–1518.

Petrie HT, Strasser A, Harris AW, Hugo P, Shortman K. 1993. $CD4^+8^-$ and $CD4^-8^+$ mature thymocytes require different post-selection processing for final development. J Immunol 151:1273–1279.

Rosenzweig M, Connole M, Forand-Barabasz A, Tremblay M-P, Johnson RP, Lackner AA. 2000. Mechanisms associated with thymocyte apoptosis induced by simian immunodeficiency virus. J Immunol 165:3461–3468.

Smith L. 1987. $CD4^+$ murine T cells develop from $CD8^+$ precursors *in vivo*. Nature 326:798–800.

Stanley SK, McCune JM, Kaneshima H, Justement JS, Sullivan M, Boone E, Baseler M, Adelsberger J, Bonyhadi M, Orenstein J, Fox CH, Fauci AS. 1993. Human immunodeficiency virus infection of the human thymus and disruption of the thymic microenvironment in the SCID-hu mouse. J Exp Med 178:1151–1163.

Su L, Kaneshima H, Bonyhadi M, Salimi S, Kraft D, Rabin L, McCune JM. 1995. HIV-1-induced thymocyte depletion is associated with indirect cytopathogenicity and infection of progenitor cells *in vivo*. Immunity 2:25–36.

Uehara S, Hitoshi Y, Numata F, Makino M, Howard M, Mizuochi T, Takatsu K. 1994. An IFN-γ-dependent pathway plays a critical role in the pathogenesis of murine immunodeficiency syndrome induced by LP-BM5 murine leukemia virus. Int Immunol 6:1937–1947.

Wang F-X, Xu Y, Sullivan J, Souder E, Argyris EG, Acheampong EA, Fisher J, Sierra M, Thomson MM, Najera R, Frank I, Kulkosky J, Pomerants RJ, Nunari G. 2005. IL-7 is a potent and proviral strain-specific inducer of latent HIV-1 cellular reservoirs of infected individual on virally suppressive HAART. J Clin Investig 115:128–137.

Woo JC, Dean GA, Pedersen NC, Moore PF. 1997. Immunopathologic changes in the thymus during the acute stage of experimentally induced feline immunodeficiency virus infection in juvenile cats. J Virol 71:8632–8641.

Wu L, Scollay R, Egerton M, Pearse M, Spangrude GJ, Shortman K. 1991. CD4 expressed on earliest T-lineage precursor cells in the adult murine thymus. Nature 349:71–74.

